# Belongingness in Medical Student Placements: Validation and Pilot Study of the Use of the Exeter Belongingness Assessment Tool in Belgian and English Medical Students

**DOI:** 10.1177/23821205241298589

**Published:** 2024-12-13

**Authors:** Rob Daniels, Thomas Pattyn, Birgitte Schoenmakers, Eric Buramba, Kato Denis

**Affiliations:** 1 School of Health and Life Sciences, 3286 University of Exeter, Exeter, England; 2 Dept of Radiology, 26657 KU Leuven, Leuven, Belgium; 3 Dept of Public Health and Primary Care, 26657 KU Leuven, Leuven, Belgium; 4 National Institute of Statistics of Rwanda, Kigali, Rwanda

**Keywords:** belongingness, legitimate peripheral participation, medical students, Belgium, United Kingdom, Exeter Belongingness Assessment Tool

## Abstract

**BACKGROUND:**

Belongingness is an important factor in the social development of medical students, and the ability to quantify belongingness in medical students may provide additional metrics by which we can compare different learning environments to help explore differential attainment. Previous studies looking at the measurement of belongingness have demonstrated good internal and external validity for tools designed to measure this facet of student experience. This study aimed to explore the use of the Exeter Belongingness Assessment Tool (EBAT) as one potential source of evidence in the study of student learning experience on clinical placements, which could be used to support quality assurance of clinical learning. This study sought to validate the use of the EBAT and carry out an initial pilot study to compare levels of belongingness in medical students in Belgium and the United Kingdom.

**METHODS:**

This study used a validated assessment tool self-administered via an online survey platform in undergraduate medical students in all years studying in Belgium and the United Kingdom.

**RESULTS:**

The EBAT described here demonstrated good internal validity in undergraduate medical students in the United Kingdom and Belgium and identified statistically significant differences between these medical student populations**.**

**CONCLUSIONS:**

These results suggest that belongingness in undergraduate medical students varies between different demographic groups and provides further evidence that the EBAT described here is a valid tool to study this. It also supports the proposal that this may be a useful tool to monitor teaching environments.

## Introduction: What Is Belongingness and How Does It Contribute to the Development of Medical Students?

Medical student selection requires the achievement of very high academic standards, yet students can struggle to achieve desired learning outcomes on clinical placements despite similar teaching. A better understanding of the affective components of their learning experience may help shed light on differential attainment and identify areas for improvement.

The journey from new medical school entrant to qualified doctor involves several transitions. The first occurs when students acquire the initial identity of “medical student,” traditionally taught separately from other students, with initiation rites and rituals. Later when they complete their undergraduate studies, they transition again to become doctors, formally entering a profession with defined knowledge, skills, and attitudes, in a similar manner to that described by Tajfel.^1^

To successfully achieve these transitions, medical students need to acquire the knowledge, skills, and attitudes of doctors required by their professional identity. A key component of this developmental process is an apprenticeship phase where students work in a clinical capacity alongside qualified doctors, with defined roles and responsibilities. These clinical environments contain the 3 key characteristics of a community of practice (domain, community, and practice) as described by Wenger and Trayner^2^ and are the cornerstone of clinical teaching in many medical schools.

Membership of these communities of practice plays a key role in the development of professional identity, which underpins professional behaviors, career success, and mental health. Membership also implies a mutual commitment, with members feeling a sense of belonging to the community of practice and in return feeling they have a legitimate role in that community. Studies of final year medical students have confirmed the importance of the apprenticeship role for development of cognitive centrality, in-group affect and in-group ties, the 3 core elements of social identity.^3^

Mirroring this process, medical students develop a sense of belongingness during their training, defined as “the need to be, and perception of being involved with others at differing interpersonal levels … which contributes to one's sense of connectedness (being part of, feeling accepted, and fitting in), and esteem (being cared about, valued and respected by others), while providing reciprocal acceptance, caring and valuing to others” as described by Levett-Jones, Lathlean.^4^ This sense of belongingness as students move through their communities of practice in clinical training is thus likely to represent an essential component of effective legitimate peripheral participation, the core function of effective communities of practice.

This sense of belongingness applies to relationships between students and their peers, between students and their training institutions and between students and the clinical teams they are placed with during placement rotations, where students need to feel valued as legitimate members of these teams with a defined role. This has been shown to play an important part in the development of professional identity through increased social capital during longitudinal clinical placements.^5^ As a result of this key role of belongingness in clinical training occurring in communities of practice, any external or internal factors that interfere with belongingness in individual students may reduce the extent to which they are able to achieve peripheral participation, impeding the development of knowledge, skills, and attitudes that are essential components of clinical training.

Tang et al have described changes in belongingness seen during curriculum and external changes during COVID lockdown^6^ and previous studies have also shown that it is possible to measure belongingness numerically in medical students.^7.8^ The study by Daniels et al^7^ in undergraduate students at the University of Exeter found that belongingness as measured by the Exeter Belongingness Assessment Tool (EBAT), was highest among final-year medical students, and there were significant differences between primary care and secondary care environments within individual students. This suggests that the clinical environment has an impact on belongingness, which can be quantified, and that it is variable, both between clinical placements and in time, peaking at graduation.^7^

Several existing tools have been developed to quantify medical student experience on attachments which include elements of belongingness, but these are either designed for and validated in different healthcare groups such as nurses (BES-CPE) or do not cover all aspects of belongingness in medical students. To facilitate study of belongingness in communities of practice, there is a need for a tool that can numerically quantify the level of belongingness in medical students in different learning environment. One of the authors of this paper (RD) has developed the EBAT that may have potential use in this context. This was developed though a review of existing placement experience assessment instruments, with themes relating to belongingness identified in the BES-CPE tool designed by Levett-Jones et al,^4^ the Manchester Clinical Placement Index,^9^ Sense of Belonging Instrument^10^ and the Dundee Ready Education Environment Measure.^11^ These formed the basis of focus group discussions with medical students at the University of Exeter resulting in development of a belongingness assessment tool designed specifically for medical students.

The resulting EBAT has 3 domains: relationship with medical school/university, relationship with peers, and experience on clinical placement. The previous study using this tool^7^ found statistically significant differences between primary and secondary care placements, and therefore the current tool has separate domains for these placements.

While the concept of numerically quantifying belongingness in undergraduate medical students was demonstrated in the study of students in Exeter by Daniels et al,^7^ this was measured at a single point in time and it remains to be determined whether it is possible to identify changes in belongingness over time, and whether different learning environments and cultures are associated with different levels of belongingness. Demonstrating that the EBAT has validity and sensitivity in this context is therefore a key step in using this approach to study belongingness in medical education.

The undergraduate medical programs in Exeter and Belgium are similar, with an initial phase of core medical sciences with some limited clinical exposure, around 14 days each year, followed by a second predominantly clinical phase in years 3 to 5. In both settings, clinical teaching in the latter years of training relies on clinical placements to develop required knowledge, skills, and attitudes expected of doctors. The main difference between the environments is that in the U.K. students typically travel to study away from home, whereas in Belgium there are fewer medical schools in a smaller geographical area, which will potentially impact social networks and thus impact on belongingness. This provided an opportunity to study the validity of the EBAT in similar populations in different countries, to determine both validity and ability to identify differences between populations of medical students, to determine the utility of the EBAT as a tool to study belongingness.

## Methods

This study set out to determine:

- Does the EBAT have statistical validity in the measurement of belongingness in medical students in Belgium and the United Kingdom
- Is the EBAT capable of identifying quantitative differences in belongingness between medical students in Belgium and the United Kingdom?

## Study design

This study used a methodological approach and psychometric analysis of the EBAT used previously in medical students in Exeter,^7^ shown in Appendix 1. This was translated into Dutch for use in the Belgian cohort by one of the authors (TP) and reviewed independently by a second (BS) who is trilingual. Due to lockdown restrictions preventing face-to-face meetings during the study period, data was collected using a self-administered prospective online survey platform^12^ with subjects recruited through social media. The questionnaire is shown in Appendix 1. Due to the challenges of conducting this research simultaneously during COVID lockdowns, a pilot study was not carried out, given the previous use of the EBAT in the same population of medical students in Exeter. Content validity of questionnaire items was confirmed by independent review of translation and relevance for the Belgian context, as described above. Sample size calculations were not carried out due to the difficulties of recruiting during lockdown conditions and requirement to use purely online sampling, rather than recruitment at scheduled face-to-face teaching sessions.

## Setting

This study was conducted on medical students in years 1 to 5 at the University of Exeter between January and April 2021, and simultaneously on students in years 1 to 5 at the Universities of Gent, Antwerp, Leuven and the Free University of Brussels in Belgium.

In total 209 Belgian medical students and 162 Exeter medical students completed the questionnaire.

## Participants and recruitment

Inclusion criteria: all undergraduate medical students registered at one of the universities at the time of the study.

### Exclusion criteria: Inability to complete the online survey

All eligible students were invited to participate through social media and email, using peer ambassadors recruited through student and teaching networks. Participation was through a shared link to an online survey platform, which collated responses automatically. All responses were anonymized, with no penalties or rewards related to participation.

## Statistical analysis

Data was collected using the online portal Online Surveys^12^ between 26th February 2021 and 17th April 2021.

Validity of the EBAT tool for the population in this study was determined according to the framework described by Downing.^13^ Evidence for content validity was achieved through the published literature on which this assessment tool was based, both individual components and the previous validation of the 39 item EBAT as described by Daniels et al.^7^ The development of this scale from draft to final product used a Delphi approach with experienced medical educationalists and experienced medical students to draft the initial scale, which was then refined after exploratory factor analysis (EFA), with low scoring items removed. This process involved a pilot study involving 145 undergraduate medical students. For the purpose of this study, validity was not assumed for either population, due to the different conditions imposed by COVID lockdowns. Data for both Belgian and U.K. students was subject to determination of internal consistency using Cronbach's alpha, and EFA with Kaiser–Meyer–Olkin (KMO) calculated, using Jamovi for statistical analysis.^14^ EFA used the Kaiser criterion,^15^ with factors retained with eigenvalues >1 and Cattrell's scree test^16^ to determine factors retention. We identified a target KMO value of 0.8 or above to identify adequate sampling and target Cronbach’s alpha of 0.7 or above, as recommended by Tavakol and Dennick.^17^

## Reporting

The reporting of this study conforms to the DoCTRINE Guidelines^18^ statement (Appendix 2).

## Results

Characteristics of respondents are shown in Table 1.

**Table 1.** Characteristics of respondents.

	U.K. STUDENTS	BELGIAN STUDENTS
Gender	Sample (%)	Sample (%)
Male	114 (70.4)	147 (70.3)
Female	42 (25.9)	62 (29.7)
Year of study		
1	32 (19.8)	4 (1.9)
2	47 (29.0)	30 (14.4)
3	23 (14.2)	51 (24.4)
4	27 (16.7)	122 (58.4)
5	29 (17.9)	2 (1.0)
Not given	4 (2.5)	
First language English		
Yes	137 (84.6)	
No	21 (13.0)	
Not given	4 (2.5)	
First language Dutch		
Yes		204 (98.1)
No		4 (1.9)

## Validation of the EBAT in Belgian Students and Comparison of the U.K. and Belgian Medical Students

### Background data

In total 371 students completed the survey in 2021: 209 (56.3%) Belgian students and 162 (43.7%) U.K. students. Students were recruited by year group student ambassadors in the United Kingdom through online forums and cohort teaching events. In Belgium, students were recruited through online forums.

### Validity of the EBAT

For both the Belgian and the U.K. survey, the belongingness tool comprising 39 questions showed a satisfactory overall internal consistency (Cronbach's alpha = 0.935 and 0.9308, respectively). For both datasets together, the internal consistency was also high with a Cronbach's alpha of 0.9310. Deducted from the questionnaire, questions are grouped in 3 groups: group peer score (q1-16), group secondary care score (q17-29), and group primary care score (q30-42).

EFA of the combined data set was tested and proved sufficient (KMO test 0.9). A principal component analysis (PCA) was performed on the 39 items and withheld 29 items grouped in 3 components (tolerable loading set at 0.5 and eigenvalues to be retained >1) after removing items that deviated from the groups (q1, q2, q3, q4, q9, q13, q24, q25, q37, and q39). A PCA with parallel analysis delivered 35 questions grouped on 3 components (excluding q9, q13, q25, and q38).

### Differences between belongingness in Belgium and the United Kingdom

The belongingness score in the combined UK–Belgium data set was normally distributed for secondary care score (*n* = 371, Shapiro Wilk test *p* > .05) but not for the peer score or primary care score. Results for each area of analysis are shown in Tables 2 , 3 and 4

**Table 2.** Comparison of U.K. and Belgian students: All students.

	BELGIAN STUDENTS	U.K. STUDENTS	DIFFERENCE (*P*-value)
Peer/university score	48.9	45.2	3.7 (.0000)
Primary care score	53.7	51.9	1.8 (.0324)
Secondary care score	44.0	46.1	2.1 (.0248)
Total score	146.7	143.2	3.5 (.0828)

**Table 3.** Comparison of U.K. and Belgian students: Male students.

	BELGIAN STUDENTS	U.K. STUDENTS	DIFFERENCE (*P*-value)
Peer/university score	49.4	43.6	5.8 (.0004)
Primary care score	55.2	49.9	5.3 (.0025)
Secondary care score	46.6	44.8	1.8 (.0886)
Total score	151.1	138.3	12.8 (.0022)

**Table 4.** Comparison of U.K. and Belgian students: Female students.

	BELGIAN STUDENTS	U.K. STUDENTS	DIFFERENCE (*P*-value)
Peer/university score	48.7	45.8	2.9 (.0023)
Primary care score	53.1	52.7	0.4 (.6110)
Secondary care score	43.0	46.5	3.5 (.0008)
Total score	144.8	145.0	0.2 (.9462)

When individual year groups were compared, in year 2, Belgian students had higher scores for total belongingness (mean score 151.8 vs 141.4; *P* = .03), primary care (mean score 54.9 vs 49.7, *P* = .02) and peer belongingness (mean score 50.9 vs 45.6; *P* = .001). In year 3, 4, and 5, there were no differences in belongingness between Belgium and U.K. students.

## Discussion

The results described here support the argument that the EBAT described here:

- Has satisfactory statistical validity in undergraduate medical students in Belgium and United Kingdom
- Identified statistically significant differences in belongingness between these groups

## Psychometric Analysis

This was the first time that the EBAT had been used in medical students who did not speak English as their first language. Given the nature of belongingness, with cultural and contextual influences, it was important in this study to confirm validity of the EBAT when translated into Dutch. The translation was carried out by a trilingual author (TP), and confirmed by a second trilingual author (BS) and then underwent factor analysis of Belgian and U.K. responses separately and then combined, to confirm internal validity. This study used the framework described by Downing^13^ to explore the argument for validity in the populations studied. The argument for content validity was addressed through analysis of published literature on components of this tool, the subsequent Delphi process and the previous validation in Exeter students described by Daniels et al.^7^ Given the very different conditions experienced by students living under lockdown compared to 2019 when the previous analysis took place in Exeter, validity was not assumed for either population. During the planning phase of this project, we identified criteria for adequate sampling and internal consistency with KMO value of 0.8 or above to identify adequate sampling and target Cronbach’s alpha of 0.7 or above, prior to the data collection. The results support the argument for validity with Cronbach's alpha = 0.935 and 0.9308 and KMO test 0.9. In addition, factor analysis supported a 3-factor structure in both populations, consistent with the structure of the EBAT.

These results provide support for the argument that despite cultural, language, and curricular differences, that the EBAT has acceptable validity under the conditions at the time. Further work to explore the internal validity under normal conditions, with a higher participation rate in Belgium would be useful to determine if the EBAT can identify changes in students over time, as living and educational environments change. A parallel study^19^ compared the Exeter scores in 2019 and 2021 reported in this study, and identified significant changes in components of belongingness, suggesting that in Exeter students at least, this tool may be useful in studying the impact of changes in learning environments on belongingness.

While this study sought to assess validity of the EBAT to measure belongingness in medical students, it did not seek to explore the correlation between academic performance and belongingness. Further work to explore the correlation between these factors would shed further light on whether belongingness as measured by this tool, has use as one of the suite of indicators of effective clinical learning. In addition, although the data reported by Daniels et al^19^ on changes in belongingness in Exeter students before and during lockdown suggest this may be a state rather than a trait, longitudinal studies looking at individual students as they move through their curriculum would be useful to explore this further, as it may be that struggling students experience a low sense of belongingness because of their poor performance rather than vice versa.

## Comparison of Belongingness in Medical Students in Belgium and the United Kingdom

Although this pilot study included a relatively small number of respondents from Belgian medical schools, due to the challenges of operating under COVID lockdown restrictions, this pilot study identified statistically significant differences between some groups or students suggesting future value as a tool to study the quality of clinical teaching environments. While the total belongingness score and primary care score did not significantly differ between U.K. and Belgian students, secondary care belongingness scores were higher in U.K. students, while belongingness among peers was significantly higher in Belgium. This was unexpected, as both countries have a traditional model of medical schools within universities, with strong group identity. One possible explanation is that the relatively small size of Belgium, with medical schools in smaller university towns, means that most students know or are distantly related to each other, with these preexisting peer relationships leading to more supportive peer groups.

An alternative explanation is that this may reflect differences in students selected for medical school, with differing ratios of students from less advantaged backgrounds, and the lower numbers of students in Exeter who reported English as a first language (84.6% vs 98.1% for Dutch as a first language in Belgium) suggest there may be demographic differences that may explain some of this difference. Previous studies^20^ have suggested a link between belongingness and academic outcomes in students who speak English as a second language, and that academic outcomes improve when measures are put in place to improve belongingness. Data from the U.K. Department of Education^21^ also suggest an association between English as an additional language and deprivation, which may have contributed to these results.

The differences reported here between male and female students merit further exploration. These may reflect differences in the extent to which working conditions in secondary care in each health system are perceived to be family friendly, resulting in Belgian hospitals having different proportion of men in senior positions, with female students feeling a lesser sense of belongingness. Medical workforce data for Belgium for 2019 show that 41.4% of primary care specialists were female, compared with 10.3% of orthopedic surgeons and 20.5% of cardiologists. In the United Kingdom in 2019, 47.6% of hospital specialists were female, similar to the overall sex ratio in medicine.^22^

The difference in the secondary care score for U.K. students in favor of female students, and in Belgian students in favor of male students warrants further exploration to identify whether implicit gender bias exists in hospital culture or training programs. The findings in Belgian students are consistent with previous studies of the career preferences of Dutch medical students^23,24^ which suggest that female gender is positively associated with general practice, psychiatry and pediatrics, while male gender was associated with secondary care specialties.

## Strengths and Weaknesses and Areas for Further Study

This study took place during national lockdowns in Belgium and Exeter, which resulted in almost all teaching taking place online with many students living off campus at the time of the data collection. There were very limited opportunities for students to socialize and most communication between students used social media. As a result, recruitment numbers were low compared to other studies, with a low proportion of students from ethnic minority groups. Previous work^25^ suggests that low measures of belongingness have a disproportionate impact on educational attainment in these students, so further studies in these students would shed more light on this.

The challenges of recruiting during COVID restrictions resulted in a particularly low response rate for some years of Belgian medical students, with an overall response around 5.9%. The inclusion of students from 3 medical schools may also have masked variation between student groups, so further studies recruiting higher numbers in individual schools are required to explore these findings. Although the clinical exposure for students in preclinical years in both countries is low, with a few sessions a year, the authors have identified differences in belongingness between primary and secondary care placements in first- and second-year students in Exeter,^7^ which is consistent with the hypothesis that teaching environment has an effect on student belongingness.

Although teaching format, content, and environment were very different from usual procedure, the primary purpose of this belongingness assessment tool is to quantify belongingness objectively, allowing the comparison of different clinical teaching environments and methods. This study demonstrated validity in the unusual conditions prevailing at the time, suggesting it has potential for the intended use. Further research in students under normal conditions is needed to further explore this. The parallel study^19^ comparing scores from students in Exeter in 2019 and 2021 has also shown statistically significant changes in belongingness over the period, supporting the argument that this tool has validity and is sensitive to changes in belongingness.

Research into teaching quality and teaching environment are prone to bias. Two of the researchers in this project (BS and RD) are involved in design and delivery of primary care teaching. Using a quantitative approach can reduce but not eliminate bias in this respect, and the researchers were cognisant of this risk during analysis, with statistical analysis being carried out independently.

## Implications

This study confirmed that the EBAT described here quantified belongingness and identified differences between different student populations. Further research into the correlation between belongingness and academic success is required to test the assertion that belongingness (and by extension legitimate peripheral participation) is a prerequisite for effective learning. Longitudinal study of individual students would also be helpful to determine the extent to which belongingness varies in individual students as they progress through different placements and explore the utility of this tool as a potential monitor of student experience.

## Conclusion

The belongingness assessment tool described in this paper demonstrated good internal validity and identified statistically significant differences between students in Belgium and the United Kingdom, suggesting potential use to compare different clinical teaching environments. This may help shed light on why some clinical teaching environments are more effective than others, and to understand the long-term effects of lockdown-related changes to teaching delivery on student development. Further work is needed to relate belongingness to academic progress.

## Supplemental Material

sj-docx-1-mde-10.1177_23821205241298589 - Supplemental material for Belongingness in Medical Student Placements: Validation and Pilot Study of the Use of the Exeter Belongingness Assessment Tool in Belgian and English Medical Students Supplemental material, sj-docx-1-mde-10.1177_23821205241298589 for Belongingness in Medical Student Placements: Validation and Pilot Study of the Use of the Exeter Belongingness Assessment Tool in Belgian and English Medical Students by Rob Daniels, Thomas Pattyn, Birgitte Schoenmakers, Eric Buramba and Kato Denis in Journal of Medical Education and Curricular Development

sj-docx-2-mde-10.1177_23821205241298589 - Supplemental material for Belongingness in Medical Student Placements: Validation and Pilot Study of the Use of the Exeter Belongingness Assessment Tool in Belgian and English Medical Students Supplemental material, sj-docx-2-mde-10.1177_23821205241298589 for Belongingness in Medical Student Placements: Validation and Pilot Study of the Use of the Exeter Belongingness Assessment Tool in Belgian and English Medical Students by Rob Daniels, Thomas Pattyn, Birgitte Schoenmakers, Eric Buramba and Kato Denis in Journal of Medical Education and Curricular Development
