# Supplementary material for: Belongingness in Medical Student Placements: Validation and Pilot Study of the Use of the Exeter Belongingness Assessment Tool in Belgian and English Medical Students
Source: J Med Educ Curric Dev. 2024 Dec 13;11:23821205241298589. doi: 10.1177/23821205241298589 (PMC11638998; doi:10.1177/23821205241298589)
Supplement: sj-docx-1-mde-10.1177_23821205241298589 - Supplemental material for Belongingness in Medical Student Placements: Validation and Pilot Study of the Use of the Exeter Belongingness Assessment Tool in Belgian and English Medical Students [file sj-docx-1-mde-10.1177_23821205241298589.docx]

**Defined Criteria To Report INnovations in Education (DoCTRINE)**

*Citation:* Blanco M, Prunuske J, DiCorcia M, Learman LA, Mutcheson B, Huang GC. The DoCTRINE Guidelines: Defined Criteria To Report INnovations in Education. Academic Medicine. 2022 May 1;97(5):689-695.

| **Introduction** | **Y/N** |
| --- | --- |
| 1. Need for the curriculum | Y |
| 1. Review of relevant literature, theories, models, or published curricula | Y |
| 1. Unique contribution of the curriculum to the literature | Y |
| **Curriculum Development** | **Y/N** |
| 1. Purpose/goals of the curriculum | Y |
| 1. Outcome-based learning objectives | Y |
| 1. Target population of learners | Y |
| **Curriculum implementation** | **Y/N** |
| 1. Instructional setting for curriculum delivery | Y |
| 1. Resources for implementing the curriculum | Y |
| 1. Description of instructional methods | Y |
| 1. Methods to evaluate achievement of outcome-based learning objectives | Y |
| 1. Origin of evaluation instrument(s) | Y |
| **Results** | **Y/N** |
| 1. Number of learners participating in the curriculum | Y |
| 1. Number of participants included in the evaluation | Y |
| 1. Evidence of achievement of outcome-based learning objectives | Y |
| **Discussion** | **Y/N** |
| 1. Summary of findings | Y |
| 1. Interpretation of findings in relation to the existing literature | Y |
| 1. Lessons learned from the implementation of the curriculum | Y |
| 1. Limitations of the evaluation of the curriculum | Y |
| 1. Describes future implications of the curriculum | Y |
